# HIF-1 expression is associated with CCL2 chemokine expression in airway inflammatory cells: implications in allergic airway inflammation

**DOI:** 10.1186/1465-9921-13-60

**Published:** 2012-07-23

**Authors:** Guillermina J Baay-Guzman, Ilona G Bebenek, Michelle Zeidler, Rogelio Hernandez-Pando, Mario I Vega, Eduardo A Garcia-Zepeda, Gabriela Antonio-Andres, Benjamin Bonavida, Marc Riedl, Eric Kleerup, Donald P Tashkin, Oliver Hankinson, Sara Huerta-Yepez

**Affiliations:** 1 Hospital Infantil de Mexico, Federico Gomez, Unidad de Investigacion en Enfermedades Oncologicas, Dr. Marquez No 262, Col. Doctores, Delegacion Cuahutemos, Mexico City, Mexico CP. 06720; 2 Facultad de Medicina Programa de Postgrado; Doctorado en Ciencias Biomedicas UNAM, Mexico City, Mexico; 3 Department of Pathology and Laboratory Medicine, David Geffen School of Medicine at UCLA, Los Angeles, CA, USA; 4 ChemRisk, LLC, Aliso Viejo, CA, USA; 5 Department of Clinical Immunology and Allergy, David Geffen School of Medicine, UCLA, Los Angeles, CA, USA; 6 Experimental Pathology Section, Department of Pathology, National Institute of Medical Science and Nutrition, Salvador Zubiran, Mexico City, Mexico; 7 Unidad de Investigacion Medica en Enfermedades Oncologicas, CMN SXXI, IMSS, Mexico City, Mexico; 8 Departamento de Inmunología, Instituto de Investigaciones Biomédicas, UNAM, México, Mexico; 9 Department of Medicine, David Geffen School of Medicine, UCLA, Los Angeles, CA, USA; 10 Department of Microbiology Immunology and Molecular Genetics, UCLA, Los Angeles, CA, USA; 11 Pulmonary and Critical Care Medicine, David Geffen School of Medicine, UCLA, Los Angeles, CA, USA

**Keywords:** Allergic airway inflammation, Asthma, Hypoxia inducible factor-1, CCL2, Arylhydrocarbon receptor nuclear translocator

## Abstract

**Background:**

The pathogenesis of allergic airway inflammation in asthmatic patients is complex and characterized by cellular infiltrates and activity of many cytokines and chemokines. Both the transcription factor hypoxia inducible factor-1 (HIF-1) and chemokine CCL2 have been shown to play pivotal roles in allergic airway inflammation. The interrelationship between these two factors is not known. We hypothesized that the expression of HIF-1 and CCL2 may be correlated and that the expression of CCL2 may be under the regulation of HIF-1. Several lines of evidence are presented to support this hypothesis.

**Methods:**

The effects of treating wild-type OVA (ovalbumin)-sensitized/challenged mice with ethyl-3,4-dihydroxybenzoate (EDHB), which upregulate HIF, on CCL2 expression, were determined. Mice conditionally knocked out for HIF-1β was examined for their ability to mount an allergic inflammatory response and CCL2 expression in the lung after intratracheal exposure to ovalbumin. The association of HIF-1α and CCL2 levels was also measured in endobronchial biopsies and bronchial fluid of asthma patients after challenge.

**Results:**

We show that both HIF-1α and CCL2 were upregulated during an OVA (ovalbumin)-induced allergic response in mice. The levels of HIF-1α and CCL2 were significantly increased following treatment with a pharmacological agent which upregulates HIF-1α, ethyl-3,4-dihydroxybenzoate (EDHB). In contrast, the expression levels of HIF-1α and CCL2 were decreased in the lungs of mice that have been conditionally knocked out for ARNT (HIF-1β) following sensitization with OVA when compared to levels in wild type mice. In asthma patients, the levels of HIF-1α and CCL2 increased after challenge with the allergen.

**Conclusions:**

These data suggest that CCL2 expression is regulated, in part, by HIF-1 in the lung. These findings also demonstrate that both CCL2 and HIF-1 are implicated in the pathogenesis of allergic airway inflammation.

## Background

Asthma is a global health concern with increasing prevalence of morbidity and mortality worldwide [1]. Asthma is a chronic inflammatory disease resulting in the overproduction of mucus, airflow obstruction, and airway hyperresponsiveness. The pathogenesis of asthma includes an exaggerated immune response to an allergen. This, in turn, activates CD4 T helper-2 (Th2) lymphocytes and the release of specific cytokines (including IL-4, IL-5, and IL-13) and chemokines (including CCL2, CCL3, CCL4, CCL5 CCL11 and CCL24) [2,3]. These cytokines and chemokines induce increased airway inflammation, including eosinophil recruitment [4].

Monocyte chemotactic protein 1 (MCP-1/CCL2) is a member of the CC family of chemokines [5], a potent chemoattractant for macrophages [6], and also attracts CD4+ and CD8+ T-lymphocytes [7]. Increasing evidence suggests that CCL2 and its haematopoietic cell receptor CC chemokine receptor 2 (CCR2) are involved in inflammatory disorders of the lungs [7]. CCL2 may have a significant role in the pathogenesis of asthma because of its ability to recruit eosinophils and monocytes, activate basophils and mast cells and induce the release of leukotriene C4 into the airway, all of which contribute to airway hyperresponsiveness [8]. CCL2 can also direct undifferentiated T-lymphocytes towards interleukin-4 (IL-4)-producing Th2 cells [9,10]. Overexpression of CCL2 has been observed in the bronchial epithelium of asthmatic patients [11]. CCL2 is also significantly upregulated after challenge in asthmatic patients [12]. Sensitization and challenge with cockroach Ag in mice, in which CCL2 activity was blocked with a specific anti-murine MCP-1 antibody, showed a decrease in several allergic inflammatory manifestations such as histamine and LTC4 levels, and attenuated airway hyperreactivity in sensitized mice [13]. Additionally, CCR2−/− mice have an attenuated airway hyperreactive response during allergen challenge or after direct instillation of CCL2, implicating a CCR2-mediated mechanism. Furthermore the neutralization of CCL2 during the allergic airway response decreased histamine in the BAL. [8]. These results support an important role for CCL2 in the asthmatic response*.*

CCL2 is known to be regulated by oxidative stress, cytokines and growth factors. In addition, CCL2 is also associated with hypoxic regulation. CCL2 was induced by both hypoxia and CoCl_2_ in human astrocytes and the promoter of CCL2 contains hypoxia response elements (HREs) which can bind hypoxia inducible factor-1 (HIF-1) [14].

The transcriptional response to hypoxia is primarily mediated by the hypoxia inducible factor (HIF) family of transcription factors. HIFs are heterodimeric proteins containing one α subunit and one β subunit. HIF-1α and HIF-2α (collectively called the HIF-α subunits) are both expressed widely, as is HIF-1β (also called the Aryl Hydrocarbon Receptor Nuclear Translocator [ARNT]). All of the HIF subunits belong to the basic helix-loop-helix (bHLH)/Per-ARNT-Sim (PAS) domain family of transcription factors. The β subunit is constitutively expressed, and the regulation of HIFs occurs mainly via effects on the α subunits. Under normoxic conditions, the HIF-α subunits are hydroxylated on key proline residues, which allows for their recognition by the von Hippel-Lindau (pVHL) tumor suppressor protein, the substrate recognition component of an E3 ubiquitin ligase complex that targets HIF-α for proteasomal degradation. Hydroxylation of both prolines is catalyzed by a family of three prolyl hydroxylases. Another level of HIF-1α regulation occurs through the hydroxylation of an asparagine residue by Factor Inhibiting Hypoxia Inducible Factor-1α (FIH) which prevents HIF-1α from interacting with the coactivator p300 under normoxic conditions [15,16]. During hypoxia, prolyl hydroxylases are inactive allowing HIF-1α to dimerize and form a complex with its partner ARNT and then binds HREs in the promoter regions of target genes, upregulating their transcription [17].

HIF-1α can be upregulated during inflammation as a consequence of the hypoxic microenvironment or via a hypoxia-independent mechanism [18]. HIF-1α can also regulate the expression of several relevant factors, including pro-inflammatory cytokines, chemokines, and adhesion molecules. Enhanced levels of HIF-1α, 2α and VEGF have been identified in lung tissues and bronchial epithelial cells of asthmatic patients as well as in the bronchial lavage of asthmatic patients, where they were associated with elevated levels of eosinophil counts as compared to persons without asthma [19]. Moreover, a direct correlation was found between the levels of HIF-1α, 2α and VEGF [19].

We recently reported that HIF-1α is increased after allergic challenge in asthma and rhinitis patients [20,21]. However, the relationship between HIF-1 and CCL2 in allergic airway inflammation has not yet been described. In this study we found a direct association between HIF-1α and CCL2 expression in allergic airway inflammation in an animal model. CCL2 was upregulated *in vivo* after treatment with a HIF-1α inducer. In contrast, CCL2 was downregulated in mice deficient in ARNT, an obligatory subunit necessary for HIF-1 activity. In addition, we also found a direct correlation between upregulated levels of CCL2 and HIF-1 after challenge in samples from asthmatic patients. These findings demonstrate for the first time, a role for HIF-1α in the regulation of CCL2 expression in airway inflammatory disease.

## Methods

### Breeding and genotyping of mice

The original Arnt^F^ allele contained a neo cassette, which was excised as described previously [22]. The Arnt^F/F^ mice, which were of a mixed C57BL/6, 129/Sv and FVB/N genetic background, were back crossed to homozygous Mx1-Cre + mice in a C57BL/6 genetic background (Jackson Laboratory, Bar Harbor, Maine). Progeny from this cross were then backcrossed at least ten successive times to generate a mouse strain in a 100% C57BL/6 background. Genotyping of the Arnt^F^ and Arnt^Δ^ alleles was performed by PCR as described previously [23]. The Mx1-Cre transgene was genotyped with PCR primers directed at the Cre gene as described previously [23]. Mx1-Cre+/- heterozygotes could not be distinguished from Mx1-Cre+/+ homozygotes by this procedure, and these genotypes are collectively referred to as Mx1-Cre+. KO mice were obtained and maintained in the facilities of the University of California, Los Angeles (UCLA) (USA).

Balb/c male mice between 6 to 8 weeks old were obtained and maintained in the facilities of the Instituto Nacional de Ciencias Medicas y de la Nutrición “Salvador Zubirán” (INCMN) (Mexico City). Mice were maintained in a pathogen-free environment, in a temperature-controlled room with 12-h dark/light cycles, and allowed food and water *ad libitum*. All experiments were performed in accordance with UCLA and INCMN animal regulations.

### Treatment of mice

Induction of the Cre protein and,thus, deletion of the Arnt allele in Mx1-Cre:Arnt^F/F^ mice was induced by intraperitoneal (i.p) injection with 500 μg of polyinosinic-polycitidylic acid (pIpC, Sigma, St.Louis, MO) in PBS on three consecutive times two days apart. Sensitization was elicited by two i.p. treatments, five days apart, with 100 μg of chicken egg ovalbumin (OVA, grade V, Sigma, St. Louis, MO) emulsified in 1 mg of aluminum hydroxide (Pierce Chemical, USA) in a total volume of 100 μL. Mice were challenged via intratracheal (i.t.) administration of 0.75% OVA on two or three occasions as indicated. Control mice received vehicle solvent only.

Where indicated, mice were i.p. injected with 100 mg/kg of ethyl 3,4, dihydroxy benzoate (EDHB) (Sigma, St.Louis, MO) in 10% DMSO. In all cases treatment with the 10% DMSO vehicle was used as negative control. Mice were euthanized by inhalation of isofluorane. All experiments were performed in accordance with UCLA and INCMN regulations.

### Lung histology and morphometric analysis

After the mice were euthanized by exsanguination, the lungs were filled intratracheally with a fixative (absolute ethanol). The lungs were then removed and fixed with ethanol, dehydrated and embedded in paraffin. For histological examination, 4-μm sections of fixed embedded tissues were cut on a Seea Meins model rotary microtome (Germany), placed on glass slides, and deparaffinized. The slides were stained sequentially with hematoxylin and eosin (H&E) to assess inflammatory cell infiltration. The area (μm^2^) of inflammation was calculated from venules with diameters of 100-200 μm and from bronchioli 150-300 μm in diameter. Four blood vessels or bronchioli from each of four to six lung sections were analyzed for each experimental group, and results are expressed as the average and standard deviations of the infiltrated area. Mucus production was assessed in lung sections stained with periodic acid Schiff (PAS), focusing on bronchioli of 150-300 μm in diameter. The PAS-positive material was measured in 200 μm squares (40,000 μm^2^). Five bronchioli from four to six lung sections were analyzed for each experimental group, and the results are expressed as the average and standard deviation of PAS-positive area (μm). Slides were analyzed under an Olympus BX-40 microscope. Quantification analysis was performed using Image Pro-plus 6.2 software (Media Cybernetics, Bethesda, MD).

### Immunohistochemistry

Deparaffinized 4-μm sections were used for immunohistochemical analysis of HIF-1α and CCL2. Briefly, antigen retrieval was performed by immersing the slides in 0.01 M sodium citrate, pH 6.0, for twentyfive minutes in boiling water. Endogenous peroxidase activity was inhibited by immersing the slides in 3% H_2_O_2_ -methanol for 20 min, and background nonspecific binding was reduced by incubating with 1% normal swine serum (NSS) in PBS for 60 min. The slides were incubated overnight at room temperature with antibodies against HIF-1α (1:250 dilution, Santa Cruz Biotechnology, Santa Cruz, CA), and CCL2 (1:500 dilution, Santa Cruz). Finally, the slides were washed five times in PBS 1X, pH 7.4, for eight minutes. In order to reduce variability, all samples from each group were processed at the same time in a single experiment using a single batch of antibody diluted in PBS with normal swine serum. After washing, the tissues were incubated with a biotinylated secondary antibody anti-rabbit IgG (1:500) (Santa Cruz) for HIF-1α or donkey anti-goat IgG biotin conjugate (1:500 Santa Cruz) for CCL2 for 30 minutes at room temperature followed by incubation with a streptavidin-HRP for 30 minutes at room temperature, and revealed with 3,3′-diaminobenzidine tetra-hydrochloride (DAKO, Carpinteria, CA, USA). The reaction was arrested with water, and the slides were counterstained with hematoxylin. Thereafter, the tissues were washed in distilled water for five minutes, dehydrated sequentially in 70%, 90% and 100% ethanol, ethanol/xylene and xylene, and then mounted with Cytoseal-60 (Fisher Scientific, Pittsburgh, PA). Finally, the slides were analyzed under light microscopy (Olympus BX-40).

### Double-immunofluorescence

HIF-1α and CCL2 colocalization in cells from BAL fluid from asthmatic patients was confirmed using immunofluorescence. Cells from BAL fluid from asthmatic patients post challenge were fixed with 4% paraformaldehyde with 0.2% Triton X-100. After blocking (5% goat serum, 3% BSA, and 0.2% Triton X-100 in PBS), the cells were incubated with anti-HIF-1α antibody for 1 h at 4°C. After incubation, the cells were treated with an FITC-conjugated anti-rabbit antibody (Jackson Immunoresearch, PA). For CCL2 detection, the cells were treated with murine anti-CCL2 antibody and developed with phycoeritryn-conjugated anti-goat antibody (Jackson Immunoresearch, PA). After double-staining, the cells were mounted using Gel/Mount (Biomeda Corp.). Immunofluorescence was examined and photographed with a Olympus B24 microscope equipped with epifluorescence illumination.

### Bronchoalveolar lavage (BAL) of mice

After the mice were euthanized by exsanguination, the tracheas were exposed and intubated with a polyethylene catheter. The BAL fluid was collected by washing with two separate aliquots of 1 ml of sterile saline (SS) containing 2% of fetal bovine serum (FBS) through the trachea. The cells from both washes were harvested by centrifugation (500 × g for 10 min at 4 °C). CCL2 in BAL supernatant fluid was determined by ELISA.

### ELISA

The quantification of serum levels of CCL2 was performed using an ELISA protocol. Briefly, micro ELISA plates were coated with monoclonal CCL2 antibody (R&D systems, Minneapolis, MN) diluted 1:500, for 2 hours. The plates were washed three times, and then blocked with chicken egg OVA (Sigma, St.Louis, MO). The plates were then washed three times and secondary antibody (R&D systems, Minneapolis, MN), diluted at 1:1000 was added. After four hours, AP-strepavidin (Zymed, San Francisco, CA) was added at a dilution of 1:1000, and the plate was incubated for 1 hour. BluePhos (KPL, Gaithersburg, MD) was used to develop the reaction and the plate was read at 650 nm within 2 hours.

### Human subjects

Nine mild to moderate asthmatic subjects who were steroid naïve and cat allergic were studied (6 female/3 male; age 26 ± 7.0 years; mean FEV_1_ [forced expiratory volume in 1 second] of 96 ± 8%). Subjects underwent a baseline fiberoptic bronchoscopy (FOB) in which endobronchial biopsies and bronchoalveolar lavage (BAL) were obtained. Three days later, subjects underwent a naturalistic cat room challenge (previously described) [24,25] until their FEV_1_ dropped by 20% or for an hour, whichever occurred first (Fel d 1 cat antigen protein concentration was 17.2 ± 5.8 ng Fel d 1/cu meter air). Three days later, subjects again underwent FOB to obtain biopsies and BAL. Two endobronchial biopsies from a randomly selected side were obtained from subsegmental or segmental carinii and placed in cassettes in 4% paraformaldehyde, transferred to 70% ETOH and then paraffin embedded and stored until immunohistochemistry (IHC) was performed. Bronchoalveolar lavage was performed with normal saline in (sub) segments of the right middle lobe or lingula in a randomized manner. 40 and 50 ml were instilled and withdrawn by syringe aspiration, filtered through a 100 μ filter, and placed on ice. The BAL was spun down at 3000 RPM for 10 minutes, the cell pellet was resuspended in fetal bovine serum (FBS), and cytospins were made (100,000 cells per slide) and stained for cell count and differential (H&E) or fixed in 4% paraformaldehyde and stored in PBS for IHC.

All subjects signed an informed consent and study activities were approved by the Human Subject Protection Committee of the University of California, Los Angeles.

### Cytoimmunostaining

Cytospins from BAL were fixed in 10% formalin and stained for HIF-1**α** and CCL2 as described above in the immunohistochemistry protocol.

### Statistical analysis

Data are expressed as mean ± S.E.M. Statistical comparisons were performed using one-way analysis of variance followed by ANOVA. Significant differences between groups were determined using the unpaired Student’s t-test. Statistical significance was set at p < 0.05. For the correlation between HIF-1**α** and CCL2 expression in the human samples, we used Pearson’s correlation analysis.

## Results

### Induction of HIF-1α results in CCL2 overexpression

**Figure 1:**
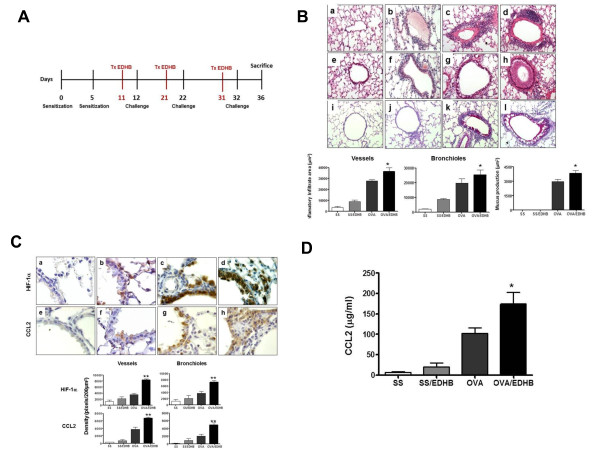
**Increased HIF expression enhances allergic airway inflammation and CCL2 levels in OVA sensitized/challenged mice. A.** Experimental Protocol. Balb/c mice were sensitized with OVA in alum, i.p., on days 0 and 5, followed by a challenge with OVA, i.t., on days 12, 22 and 32. Mice were treated with 100 mg/kg EDHB, i.p., 24 prior to each challenge. Mice were sacrificed 4 days after the last challenge, on day 36. **B**. Representative H&E stained sections of lung tissues showing blood vessels (a-d) or bronchioles (e-h) obtained from OVA-sensitized/challenged (OVA) (c and g) and OVA sensitized/challenged mice treated with EDHB (OVA/EDHB) (d and h) or control mice treated with saline (SS) (a and e) and SS/EDHB (b and f) are presented in the upper panel. Representative PAS stained sections of lung tissue from OVA-sensitized mice (k) and OVA/EDHB (l), SS (i) or SS/EDHB (j) are also presented in the top panel. Quantification of the inflammatory response and mucus production for five mice from each experimental group is presented in the bottom panel. The degree of inflammatory infiltration in venules and middle size veins 100–200 μm in diameter and in bronchioles 150–300 μm in diameter from five mice from each experimental group was determined as described in “Materials and Methods.” Results are expressed as means and standard deviations of infiltrate area. (empty) saline control; (light grey) EDHB; (grey) OVA; (solid) OVA/EDHB. *p = 0*.*01 OVA *vs.* OVA/EDHB mice. **C.** Immunohistochemical staining of HIF-1**α** (a-d) and CCL2 (e-h) expression in the lung tissue of representative mice treated with OVA (c and g), OVA/EDHB (d and h), SS (a and e) and SS/EDHB (b and f) is shown in the upper panel. Quantification of HIF-1α and CCL2 immunostaining in the vessels and bronchioles of five mice from each experimental group is presented in the lower panel. Five lung sections were analyzed for each experimental group. *p = 0.001 OVA *vs.* OVA/EDHB mice. **D**. Quantification of CCL2 in BALF of mice treated with saline (empty bar), SS/EDHB (light grey), OVA (grey) and OVA/EDHB (solid bar). *p = 0*.*002 OVA *vs.* OVA/EDHB mice.

We investigated whether upregulation of HIF-1**α** would affect CCL2 expression. We used EDHB, which inhibits prolyl hydroxylases competitively with regard to two of their cosubstrates, oxoglutarate and ascorbate [26]. Balb/c mice were sensitized with OVA, i.p., on days 0 and 5. Next, EDHB was injected, i.p. on three occasions, one day before each ntratracheal challenge with OVA, and the mice were sacrificed four days after the last OVA treatment (Figure 1A). The inflammatory response was measured in lung tissue using H&E and PAS staining (Figure 1B). OVA treatment led to an increase in perivascular (Figure 1Bc) and peibronchiolar (Figure 1Bg) inflammation. Treatment with OVA and EDHB (Figure 1Bd and 1Bh) led to a markedly enhanced inflammatory response compared with treatment with OVA alone. EDHB elicited a modest inflammatory response by itself (Figure 1Bb and 1Bf). In addition, PAS staining showed an increase in mucus production in mice treated with OVA (Figure 1Bk) as compared to saline treated mice (Figure 1Bi). Enhanced mucus production was observed after treatment with the combination of OVA and EDHB (Figure 1Bl) as compared to OVA alone. EDHB treatment alone did not induce mucus production (Figure 1Bj). Quantitation of the inflammatory response (Figure 1B bottom panel) shows that the observed differences were statistically significant.

Immunohistochemical analysis (Figure 1C) demonstrated that OVA and EDHB enhanced the expression of HIF-1α (Figure 1Cd) and CCL2 (Figure 1Ch) above the levels elicited by OVA alone (Figure 1Cc and g). VEGF expression was also significantly upregulated (data not shown) [21]. Lungs of saline-treated mice did not show significant expression of either HIF-1α or CCL2 (Figure 1Ca and e). EDHB alone slightly upregulated HIF-1α and CCL2 expression (Figure 1Cb and 1Cf). Quantification of the expression of HIF-1α and CCL2 revealed that the enhanced expression of both proteins in response to OVA and OVA and EDHB treatment was statistically significant (Figure 1C, bottom panel). Furthermore, using flow cytometry, we determined that macrophages were the principal cells expressing HIF-1α and CCL2, while B and T cells expressed lower levels of these proteins; dendritic cells expressed modest amounts of the proteins. In addition we found a higher amount of HIF-1 and CCL2 doubly-stained positive cells in OVA-sensitized mice as compared with control mice (data not shown).

Thus, during airway inflammation, CCL2 was upregulated by EDHB, an agent which upregulates HIF and at least one of its target genes, VEGF (data not shown) [21]. In addition, we analyzed the expression of CCL2 in the lavage of treated mice. We found a significant increase in the expression of CCL2 in the lavage fluid of the OVA and EDHB treated mice as compared to mice treated with OVA alone (Figure 1D).

### CCL2 expression is reduced in mice deficient in ARNT

Mice that are homozygous for an Arnt null allele (i.e. Arnt knockout mice) die in utero [27,28]. We, therefore, used conditionally knocked out mice in which deletion of the Arnt gene can be deleted in adulthood [21]. Briefly, the ARNT allele in the Arnt^F/F^:Mx1-Cre mice can be deleted with treatment with polyinosinic-polycytidylic acid (pIpC), which activates transcription of the Cre gene. The Cre recombinase catalyzes deletion of the genomic segment between two loxP sites. Thus, in Arnt^F/F^:Mx1-Cre^+^ mice i.p. injected with pIpC, inactivation of the Arnt gene occurs in most tissues of the body and at about 80% efficiency in the lung [22]. In order to test our hypothesis that HIF-1 plays a role in CCL2 expression during airway inflammation, we examined the expression of CCL2 in mice sensitized and challenged with OVA and deficient for the ARNT allele.

**Figure 2:**
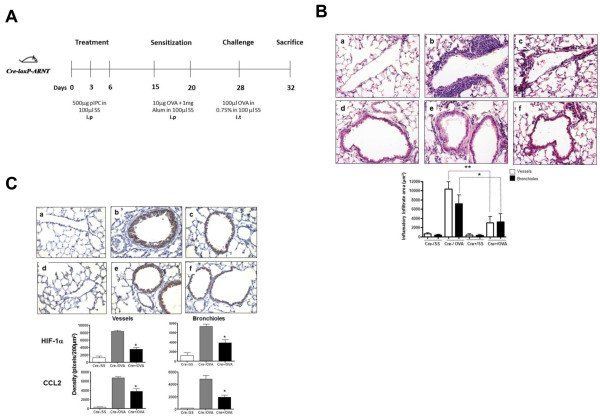
**OVA sensitized/challenged mice conditionally knocked out for Arnt have a reduced allergic response in the lung and decreased expression of CCL2. A.** Experimental protocol. Mice were treated with pIpC three times, three days apart, to delete the *Arnt* allele. In order to induce allergic airway inflammation, mice were sensitized with OVA in alum, i.p., on days 15 and 20, followed by a challenge with OVA, i.t., on day 28. Mice were sacrificed on day 32, 4 days after the challenge. **B.** Histological analyses of lung tissue. Representative sections of lung tissues were obtained from OVA-sensitized/challenged mice (b,c,e and f) and control mice treated with sterile saline (SS) (a and d), of genotype *Arnt*^*F/F*^*:Mx-1-Cre*^*–*^ (b and e) or *Arnt*^*F/F*^*:Mx1-Cre*^*+*^ (c and f). The lung tissues (a-c, blood vessels; and d-f bronchioles) were stained with H&E and examined by light microscopy (upper panel). Quantification of the inflammatory response is shown in the lower panel. The degree of inflammatory infiltration in venules and middle size veins 100–200 μm in diameter, and in bronchioles 150–300 μm in diameter from five mice from each experimental group was determined as described in “Materials and Methods.” Results are expressed as averages and standard deviations of infiltrated area. (empty bars) perivascular infiltrate; (solid bars) peribronchiolar infiltrate. *p = 0.01, **p = 0.001 Cre- OVA vs. Cre + mice. **C.** Immunohistochemical staining of HIF-1α (a-c) and CCL2 (d-f) in lung tissue of representative *Arnt*^*F/F*^*:Mx1-Cre*^*-*^ (b and e) or *Arnt*^*F/F*^*:Mx1-Cre*^*+*^ (c and f) in OVA (b,c,e and f) and saline (a and d) mice treated as described in B (upper panel). Quantification of HIF-1α and CCL2 (lower panel) was performed as described in Figure 2B. *p < 0*.*05 Cre- OVA vs. Cre + mice.

Eight Arnt^F/F^:Mx1-Cre mice per group were used in these experiments. The mice received pIpC to delete the *Arnt* gene. Mice were subjected to a previously described allergenic protocol [20], involving two intraperitoneal treatments with the allergen OVA, and the adjuvant, alum, followed by three intratracheal administrations of OVA (Figure 2A). Routine H&E stained lung sections were used to evaluate the degree of inflammatory cell infiltration (Figure 2B). OVA treatment in the Cre^-^ mice elicited a marked perivascular (Figure 2Bb) and peribronchiolar (Figure 2Be) inflammatory infiltration, comprised mostly of mononuclear cells. The Cre^+^ mice treated with OVA exhibited a marked reduction in the degree of perivascular (Figure 2Bc) and peribronchiolar (Figure 2Bf) inflammatory cell infiltration, as compared to the similarly treated Cre^-^ mice (Figure 2Bb and e). Saline treated mice did not show any significant inflammatory response (Figure 2Ba and d). The decrease in infiltration in the Cre^+^ mouse lungs was statistically significant (Figure 2B, bottom panel).

We also analyzed the expression of HIF-1α and CCL2 in the lungs of these mice. Representative lung sections from each experimental group and quantitative analysis of the staining in all the mice are presented in Figure 2C. The expression of HIF-1α and CCL2 was drastically reduced in the Cre^+^/OVA treated mice (Figure 2Cc and f), as compared to the Cre-/OVA mice (Figure 2Cb and e). OVA treatment of the Cre^-^ mice (Figure 2Cb and e) increased HIF-1α levels in the nucleus and CCL2 levels in the cytoplasm of inflammatory and bronchiolar epithelial cells as compared to untreated mice (Figure 2Ca and d). Reduction of HIF-1α and CCL2 in both vessels and bronchioles in Cre^+^/OVA mice as compared to Cre-/OVA mice was statistically significant (Figure 2C bottom panel).

### CCL2 expression correlates with increased HIF-1α expression in asthmatic patients after allergen challenge

**Figure 3:**
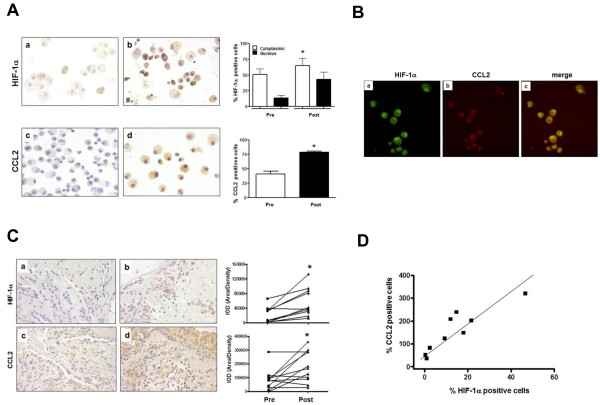
**HIF-1α and CCL2 are both increased in BALF and lung tissue after challenge in asthmatic patients. A.** Representative photomicrographs of HIF-1α (a and b) and CCL2 (c and d) immunostaining observed at magnification of 100X in epithelial cells from bronchial lavage before (a and c) and after (b and d) challenge. In the right panel, we quantified HIF-1α and CCL2 positive cells, before and after the challenge and found a significant increase in the expression of both genes. Student t-test *p < 0.05 pre *vs.* post challenge HIF-1α and CCL2 expression. **B.** The colocalization of HIF-1**α** and CCL2 in BAL cells from asthmatic patients was performed using double-immunofluorescence. The cells were incubated with anti-HIF-1**α** antibody and then with FITC-conjugated anti-rabbit antibody (a). For CCL2 detection, the cells were treated with goat anti-CCL2 antibody and then treated with a secondary antibody with red fluorescence (b). The merging of fluorescence resulted in significant yellow fluorescence that indicated co-expression of HIF-1**α** and CCL2 (c). **C**. Representative photomicrographs of HIF-1**α** (a and b) and CCL2 expression (c and d) before (a and c) and after (b and d) challenge, observed at magnification of 40X in lung tissue (left panel). Both HIF-1**α** and CCL2 expression was enhanced after allergen challenge in the lung tissue. In the right panel, we quantified the density of the expression of HIF-1**α** and CCL2 before and after challenge. This analysis was performed from data collected from 9 patients. Student t-test *p < 0.05 Pre *vs.* post challenge HIF-1**α** and CCL2 expression. **D**. Correlation analysis between HIF-1**α** and CCL2 positively immunostained cells after challenge. Pearson’s analysis (*p* = 0.001, r = 0.878) post challenge HIF-1**α***vs*. CCL2.

We also evaluated CCL2 expression in human samples (n = 9) in order to validate our findings in mice. The subjects general clinical characteristics are described in Table 1. We examined HIF-1α and CCL2 expression in bronchial lavage fluid (Figure 3A) and lung tissue (Figure 3B) derived from asthmatic patients before and after allergen challenge. Figure 3A shows representative photomicrographs of HIF-1α (Figure 3Aa and b) and CCL2 (Figure 3Ac and d) immunostained cells obtained from lavage in asthmatic patients before (Figure 3Aa and c) and after (Figure 3Ab and d) exposure to antigen challenge. HIF-1α and CCL2 expression was significantly increased in cells from lavage after allergen challenge (Figure 3A, right panel). In order to demonstrate the coexpression of HIF-1**α** and CCL2 in cells from lavage, we performed double-immunostaining (Figure 3B). Our results demonstrate that HIF-1**α** (3Ba) and CCL2 (3Bb) were colocalized in the same cells (Figure 3Bc).

**Table 1:** Clinical features of patients with asthma

**Asthmatic subjects**	
Subjects n	9
Age years	26 (18-43)
Sex M:F	3:6
FEV1% predicted	94.67 (76-112)
FEV1 (L)	3.9 (2.9-5.0)
FEV1 at 0 min (L) [CRC]	3.7 (2.6-4.9)
FEV1 Lowest (L) [CRC]	3.39 (2.5-4.3)
% FEV1 decline [CRC]	8.72 (-8.3-32.1)

In Figure 3C we show HIF-1**α** (Figure 3Ca and b) and CCL2 (Figure 3Cc and d) immunostaining of lung tissue from asthmatic patients, before (Figure 3Ca and c) and after (Figure 3Cb and d) exposure to challenge. Expression of HIF-1**α** was predominantly nuclear, although some cytoplasmic staining was observed. Expression of CCL2 was predominantly cytoplasmic or in the membrane. The right panel of Figure 3C shows quantification of the density of the expression of HIF-1**α** and CCL2 in the lung tissue. Expression of HIF-1**α** and CCL2 was significantly higher after challenge. Furthermore, there was a direct correlation between HIF-1**α** and CCL2 expression after challenge, as measured by inmunostaining (Figure 3D), (*p < 0.05, *r = 0.7930).

These findings therefore build on our animal studies and suggest that HIF-1 plays an important role in the regulation of CCL2 during allergic disease in the human.

## Discussion

Previous observations have implicated HIF-1 in the development of asthma [21]. Hypoxia is a potent stimulus for inflammation and remodeling, and HIF-1 can activate transcription of several inflammatory cytokines, chemokines and growth factors, including VEGF, as well as matrix remodeling proteins, such as procollagen and matrix metalloproteinases [29]. N-acetylcysteine amide (AD4), a newly developed antioxidant thiol compound, attenuated airway inflammation as well as hyperresponsiveness by regulating HIF-1**α** and NF-κB and reducing reactive oxygen species (ROS) levels [30]. Inhibition of PPAR decreased HIF-1 and NF-κB and induced attenuation of the airway inflammatory response [31]. VEGF and HIF-1**α** and HIF-2**α** were expressed at higher levels in asthmatic patients as compared to healthy controls [19]. Most recently, Guo et al. found a significant reduction in the number of eosinophils in BAL fluid after sensitization/challenge with OVA in mice heterozygous for a HIF-1α null allele as compared to wild-type mice [32]. However, a clear mechanism of how HIF-1 is involved in perpetuating the inflammatory response is not clear because HIF-1 regulates several chemokines and cytokines. Previous studies have demonstrated that HIF-1**α** regulates CCL2 at the transcriptional level [14]. We provide evidence for this notion in the present study in that at least one mechanism for HIF-1-mediated inflammation in the lung during an allergic response is via the upregulation of the pro-inflammatory chemokine CCL2, mainly by macrophages, which in turn, can recruit cells such as eosinophils and lymphocytes to areas of inflammation.

In a previous study conducted by our group, EDHB, a known inducer of HIF-1**α** both *in vivo* and *in vitro*[33], resulted in increased levels of HIF-1**α** and the target gene VEGF in the airway epithelium of mice [21]. EDHB also enhanced the allergenic response to OVA, including airway inflammation, goblet cell hyperplasia, and increased mucus production, indicating that HIF-1 is involved in generating the allergic inflammatory response. In order to investigate whether one of the mechanisms of HIF-1-mediated lung inflammation is via upregulation of the pro-inflammatory chemokine CCL2, we analyzed the effect of EDHB on airway inflammation in combination with OVA. Mice treated with EDHB and OVA had significantly higher expression of CCL2 than mice treated with OVA alone and these levels correlated with the expression of HIF-1**α** and the inflammatory response (Figure 1C). EDHB alone was sufficient to induce an inflammatory response in our model as previously observed [21] as well as CCL2 expression. To our knowledge, this is the first time that a compound known to induce HIF-1 activation has been shown to induce the expression of CCL2 in the absence of a specific allergenic stimulus.

In order to validate our hypothesis that HIF-1α regulates CCL2 during allergic airway inflammation, we utilized mice conditionally knocked out for ARNT, which is an obligatory subunit of HIF-1 [34]. Arnt2 is not expressed in mouse (or human) lung [22] and, therefore, its potential effects can be excluded from our study. It should be noted that both HIF-1**α** and HIF-2**α** are expressed in the mouse lung [21], particularly in the bronchiolar epithelium and lung vascular endothelium. Therefore, both **α** subunits are likely to contribute towards airway inflammation. There appears to be some redundancy between HIF-1**α** and HIF-2**α**, since they exhibit different (although overlapping) spectra of target genes [35]. Deleting each α subunit individually is likely to provide limited insight into the role of HIF-1 in the development of allergic airway inflammation. Furthermore, interpretation of data from these mice could be further confounded by the compensatory upregulation of one **α** subunit that can occur when the other is decreased or ablated [36-39]. Hence, our ARNT conditional knock out mice are well suited for this study compared to individually knocked out HIF-1**α** or HIF-2**α** mice. It should be noted that although ARNT is also an obligatory subunit for the Aryl hydrocarbon receptor (AhR), C57BL/6 *Ahr*-null mice do not exhibit a reduced allergic lung inflammatory response after an OVA challenge protocol very similar to the one we performed here [40].

Using our conditionally knocked out mice, we observed a significant decrease in HIF-1**α** expression and more importantly a decrease in CCL2 expression; this observation was associated with a decreased inflammatory response (Figure 2C). This finding clearly demonstrates that CCL2 is under the regulation of HIF-**α** and that it is implicated in the allergic inflammatory response. Our findings provide evidence for the important involvement of HIF-1**α** in the regulation of CCL2 expression in allergic inflammatory disease. Altogether, the present study suggests that this is also likely to be the case in humans, with possible significant clinical implications.

We observed significant upregulation of HIF-1**α** and CCL2 in epithelial cells from bronchial lavage and lung tissue of asthmatic patients after exposure to allergen. Although one previous study correlated HIF-1**α** and 2**α** overexpresion in bronchial lavage and lung tissue from asthmatic patients as compared to healthy controls, the levels of protein were only measured in pre-challenge conditions [19]. A recent study by our group demonstrates a significant increase of HIF-1 and its target gene VEGF after allergen challenge [21]. However, to our knowledge this is the first time that a direct correlation between HIF-1**α** and CCL2 expression has been shown in asthmatic patients after cat allergen challenge.

In the current study, we provide compelling evidence for a direct association between the expression of HIF-1 and CCL2 during allergic lung inflammation in the mouse using a combination of pharmacological and genetic approaches. We also provide evidence for this association in asthmatic patients. Our animal studies and supporting human data indicate an important role for the HIF-1α-mediated allergic inflammatory response possibly via regulation of the pro-inflammatory chemokine CCL2. Further studies will have to address the direct regulation of CCL2 by HIF-1 during allergic airway inflammation. Furthermore, although CCL2 may not be considered an ideal pharmacotherapeutic target for the treatment of asthma, our results indicate that HIF-1α may be used as a specific target.

## Competing interests

The authors declare that they have no competing interests. Funding has been received from the Collaborative Research Grant from the University of California Institute for Mexico and the United States (UC MEXUS-CONACYT) (S.H-Y and O.H). Mexico Federal Funds Grant HIM/2008/034 (S.H.-Y., G.B.-G.), National Institutes of Health grants R01 CA28868 (O.H.), R01 HL080343 (E.K. and M.Z.), NIH/NHLBI R01-HL-080343 (D.T) and NIH/NCATS UL1-TR-000124 (E.K., M.Z and D.T).

## Authors contributions

GJBG carried out the experimental models and immunostaining and performed the statistical analysis. IGB prepared the manuscript. MZ designed the human studies, and analyzed and interpreted the data. RHP designed the experimental models. MIV edited the manuscript. EGZ participated in carrying out the experimental models. GAA carried out the immunofluorescence assay. BB edited the manuscript. MR participated in the design of the human study and provided samples. EK designed the human studies and analyzed and interpreted the data. DPT participated in the design of the human study. OH participated in the design of the KO mouse study and edited the manuscript. SHY conceived the study, and participated in its design and coordination, and prepared the manuscript.

All authors read and approved the final manuscript.
